# Intratracheal Instillation of High Dose Adenoviral Vectors Is Sufficient to Induce Lung Injury and Fibrosis in Mice

**DOI:** 10.1371/journal.pone.0116142

**Published:** 2014-12-31

**Authors:** Qiyuan Zhou, Tianji Chen, Melike Bozkanat, Joyce Christina F. Ibe, John W. Christman, J. Usha Raj, Guofei Zhou

**Affiliations:** 1 Department of Pediatrics, University of Illinois at Chicago, Chicago, Illinois, United States of America; 2 Children’s Hospital University of Illinois, Chicago, Illinois, United States of America; 3 Division of Pulmonary, Allergy, Critical Care and Sleep Medicine, The Ohio State University, Columbus, Ohio, United States of America; 4 University of Illinois Cancer Center, University of Illinois at Chicago, Chicago, Illinois, United States of America; Chinese Academy of Sciences, China

## Abstract

**Rationale:**

Replication deficient adenoviruses (Ad) vectors are common tools in gene therapy. Since Ad vectors are known to activate innate and adaptive immunity, we investigated whether intratracheal administration of Ad vectors alone is sufficient to induce lung injury and pulmonary fibrosis.

**Methods:**

We instilled Ad viruses ranging from 10^7^ to 1.625×10^9^ ifu/mouse as well as the same volume of PBS and bleomycin. 14 and 21 days after administration, we collected bronchoalveolar lavage fluid (BALF) and mouse lung tissues. We measured the protein concentration, total and differential cell counts, and TGF-β1 production, performed Trichrome staining and Sircol assay, determined gene and protein levels of profibrotic cytokines, MMPs, and Wnt signaling proteins, and conducted TUNEL staining and co-immunofluorescence for GFP and α-SMA staining.

**Results:**

Instillation of high dose Ad vectors (1.625×10^9^ ifu/mouse) into mouse lungs induced high levels of protein content, inflammatory cells, and TGF-β1 in BALF, comparable to those in bleomycin-instilled lungs. The collagen content and mRNA levels of Col1a1, Col1a2, PCNA, and α-SMA were also increased in the lungs. Instillation of both bleomycin and Ad vectors increased expression levels of TNFα and IL-1β but not IL-10. Instillation of bleomycin but not Ad increased the expression of IL-1α, IL-13 and IL-16. Treatment with bleomycin or Ad vectors increased expression levels of integrin α1, α5, and αv, MMP9, whereas treatment with bleomycin but not Ad vectors induced MMP2 expression levels. Both bleomycin and Ad vectors induced mRNA levels of Wnt2, 2b, 5b, and Lrp6. Intratracheal instillation of Ad viruses also induced DNA damages and Ad viral infection-mediated fibrosis is not limited to the infection sites.

**Conclusions:**

Our results suggest that administration of Ad vectors induces an inflammatory response, lung injury, and pulmonary fibrosis in a dose dependent manner.

## Introduction

Adenoviruses (Ad) are simple double stranded DNA viruses that are nonenveloped [1]. Among approximately 50 distinct serotypes, the group C (serotype 2 and 5) has been most extensively studied and has been used as vaccine vectors and gene therapy vectors [2]. Replication deficient Ad vectors can carry large DNA, are easy to produce in large quantities, and have broad cell tropism. These features make them ideal research tools to manipulate expression of candidate genes in cell culture and/or in small animals. In the field of respiratory diseases, Ad vectors have been used to study pathogenesis of fibrosis, Chronic Obstructive Pulmonary Disease (COPD), etc. [3]–[5], where inflammation contributes to the diseases. However, Ad vectors themselves can activate innate and adaptive immunity, leading to production of inflammatory cytokines and chemokines [1]. As a result, it is difficult to distinct the effect of the target gene from the effect of Ad vector-mediated inflammation.

Idiopathic pulmonary fibrosis (IPF) is a devastating disease without cure [6], [7]. Multiple experimental models of IPF have been developed in several species with bleomycin-induced fibrosis as the most common model [8], [9]. Intratracheal administration of bleomycin causes lung injury in the early stage and pulmonary fibrosis in the late stage [10], [11]. Many studies suggest that inflammation plays a key role in the development of fibrosis [10], [11]. Furthermore, Ad vectors have been adopted to study the function of target genes, such as TGF-β1, in the pathogenesis of fibrosis [3], [12]. Given the inflammatory effect of Ad vectors, it remains unclear whether the Ad vector-mediated inflammation also contributes to pulmonary fibrosis.

In this study, we investigate whether intratracheal administration of Ad vectors alone is sufficient to induce lung injury and pulmonary fibrosis. We compared Ad vector-mediated lung injury and fibrosis to bleomycin-induced lung injury and fibrosis. We found that Ad vectors induced profibrotic cytokine production, lung injury, and fibrosis in a dose dependent manner. We also found that administration of Ad vectors induced expression of MMPs, integrins, and Wnt signaling, which are consistent with the fibrotic phenotype. Administration of Ad vectors also induced DNA damage in lungs and the fibrotic sites are not limited to the infection sites. Thus, we conclude that administration of high dose of Ad vector is sufficient to induce a bleomycin-like lung injury/fibrosis.

## Methods

### Delivery of PBS, bleomycin, and adenoviruses to mouse lungs

8- to 10-week-old pathogen-free male C57BL/6 mice (Jackson Laboratory, Bar Harbor, Maine) were treated with intratracheal instillation of 50 µl of phosphate-buffered saline (PBS), bleomycin (0.045 U), or adenoviruses that were dissolved in PBS in two aliquots (25 µl each). Animals were maintained in a 12∶12-h light-dark cycle with food and water *ad libitum.* The experimental protocols were approved by the University of Illinois at Chicago Institutional Animal Care and Use Committee and animals were handled according to National Institutes of Health guidelines and approved experimental protocols. Mice were anesthetized with ketamine 100 mg/kg/xylazine 10 mg/kg and euthanasized with cervical dislocation under anesthesia. After the experiments, mouse lung tissues were excised, snap-frozen, or inflated with 4% (w/v) paraformaldehyde in PBS, embedded in paraffin, sectioned, and stained with H&E and Masson’s Trichrome [10], [13]. The slide of the Masson’s Trichrome staining scanned on the Aperio ScanScope CS (Leica Microsystems Inc. Buffalo Grove, IL) at 20x magnification at standard resolution. The percent of tissue area that was classified as fibrosis was quantified with Aperio ImageScope v11 software (Leica Microsystems Inc. Buffalo Grove, IL).

### Adenovirus amplification and the measurement of titer

Adenovirus were packaged in 293A cells and the titer (infectious unit, ifu) was determined by Adeno-X Rapid Titer Kit following the User’s Manual (Clontech Laboratories, Inc. Mountain View, CA).

### Measurements of lung fibrosis and lung injury

Presence of inflammation and lung injury was determined by measuring protein levels and total and differential cell counts in bronchoalveolar lavage fluid (BALF) at days 14 and 21 after PBS, bleomycin, or Ad vector treatment. Each mouse was injected with 1 ml PBS and approximately 800 µl BALF was acquired from each mouse. 180 ul of BALF were applied to Thermo Scientific Cytospin 4 (Thermo Fisher Scientific, Kalamazoo, MI) at 1000 rpm for 5 minutes, followed with Wright staining and counting of the numbers of Lymphocytes, macrophages, and neutrophils. The BALF was also centrifuged at 13,000 *g* for 5 minutes, and protein concentrations in the supernatants determined with a Bio-Rad protein assay (Bio-Rad, Hercules, CA) [14]. Mouse lungs were obtained for Sircol Soluble Collagen Assay (Biocolor Ltd, County Antrim, United Kingdom) [13] and stained with H&E and Masson’s Trichrome for the assessment of fibrosis.

### Quantitative real-time RT-PCR

Total RNA was isolated using the miRNeasy mini kit (Qiagen, Valencia, CA) and quantified with Nanodrop 2000 Spectrophotometer (Thermo Scientific, Waltham, MA). After synthesis of complementary DNA (cDNA) with the ABI High Capacity cDNA Reverse Transcription Kit (Applied Biosystems Inc., Foster City, CA), quantitative real-time RT-PCR (qRT-PCR) was carried out on the ABI StepOnePlus real-time PCR system with ABI SYBR Green PCR master mix (Applied Biosystems Inc.). Cycle threshold values were normalized to the amplification of the mitochondrial ribosomal protein L19 (RPL19). The following primers were used for the real time qRT-PCR: mouse RPL19, AGCCTGTGACTGTCCATTC (sense), ATCCTCATCCTTCTCATCCAG (antisense); mouse integrin α5, AGGTGGGCAGGGTCTACATCT (sense), CGAATCGGCTGAACTCATCTT (antisense); mouse integrin αv, AGGATGGGCTTTTTCAAACGT (sense), TTCCTTCGCCATTCTCATGAG (antisense); mouse integrin α1, GAAACGAGAGCTGGCTATTCAGA (sense), AGGCGCTCAGGAGGATAACC (antisense); mouse MMP2, GGACAGTGACACCACGTGACA (sense), GGCCTCATACACAGCGTCAAT (antisense); mouse MMP9, GGACGACGTGGGCTACGT (sense), CACGGTTGAAGCAAAGAAGGA (antisense); mouse collagen type 1A1 (Col1a1), GCACGAGTCACACCGGAACT (sense), AAGGGAGCCACATCGATGAT (antisense); mouse collagen type 1A2 (Col1a2), CTACTGGTGAAACCTGCATCCA (sense), GGGCGCGGCTGTATGAG (antisense); mouse TNFα, CCCAAGGCGCCACATCT (sense), CCACGTCGCGGATCATG (antisense); mouse TGF-β1, TCGACATGGAGCTGGTGAAA (sense), CTGGCGAGCCTTAGTTTGGA (antisense); mouse TGF-β2, ACCTTTTTGCTCCTGCATCTG (sense), TGCGCATAAACTGATCCATGTC (antisense); mouse TGF-β3, TGACCCACGTCCCCTATCAG (sense), CCCCGTGCATCTCTTCCA (antisense); mouse IL-1α, GCCCGTGTTGCTGAAGGA (sense), AGAAGAAAATGAGGTCGGTCTCA (antisense); mouse IL-1β, CTACAGGCTCCGAGATGAACAAC (sense), TCCATTGAGGTGGAGAGCTTTC (antisense); mouse IL-13, GCTCAGCTACACAAAGCAACTGTT (sense), TGAGATGCCCAGGGATGGT (antisense); mouse Wnt2b, TGCCAAAGAGAAGAGGCTTAAGG (sense), CGACCACAGCGGTTGTTGT (antisense); mouse Wnt2, CACCAGTTCCGCCAGCAT (sense), GAGGACCCGGCCAAAGA (antisense); mouse Wnt5b, CCAAGACGGGCATCAGAGA (sense), CACGGTGCTGCAGTTCCA (antisense); mouse Lrp6 (lipoprotein receptor-related protein 6), TGGCTTGGCGGTGTGAT (sense), TGCCCGCTGGCACACT (antisense); mouse α-SMA (Acta2), CGGGAGAAAATGACCCAGATT (sense), GGACAGCACAGCCTGAATAGC (antisense); mouse PCNA (Proliferating cell nuclear antigen), GCCAGACCTCGTTCCTCTTAGA (sense), TCAGGCGTGCCTCAAACAT (antisense).

### Western blotting

Mouse lungs were lysed in 150–250 µl of mRIPA buffer (50 mM Tris at pH 8.0, 150 mM NaCl, 1% NP-40, 1% sodium deoxycholate, and protease inhibitors) and cell lysates were cleared by centrifugation at 13,000 *g* for 5 minutes. Protein concentrations of the supernatants were determined with a Bio-Rad protein assay (Bio-Rad, Hercules, CA). Proteins were then separated by SDS-polyacrylamide gel electrophoresis and transferred to BA-S 85 nitrocellulose membrane (OPTITRAN, Middlesex, UK). Proteins were detected with Supersignal West Pico Chemiluminescent Substrate (Thermo Scientific, Rockford, IL). Gray density of Western blots was measured using ImageJ software (National Institutes of Health, Bethesda, MD). The following antibodies were used in this study: integrin α5 (Santa Cruz Biotech, Santa Cruz, CA), MMP9 (Cell Signaling, Danvers, MA), and Wnt 5b (Sigma-Aldrich, St. Louis, MO).

### Measurement of TGF-β, IL-1β, and TNFα in BALF

Levels of TGF-β (R&D Systems, Minneapolis, MN), IL-1β (Thermo Scientific, Rockford, IL), and TNFα (BD Biosciences, San Jose, CA) were measured using an ELISA kit following the manufacturer’s protocol. Briefly, we coated a 96-well microplate with capture antibody overnight at room temperature. After three washes with wash buffer (0.05% Tween-20 in PBS), we blocked each well of the plate with the blocking buffer (5% Tween 20 in PBS with 0.05% NaN_3_) for 1 hour before we added aliquots of BALF (5 ul) or standards and incubated for additional 2 hours at room temperature. After three washes, we added the detection antibody and incubated plates for 2 hours, followed with the addition of the working dilution of streptavidin in the dark for 20 minutes. After three washes, we added the substrate solution, incubated the plate for 20 minutes, and stopped the reaction with the stop solution. The optical density of each well was determined immediately using a GloMax-Multi Detection System (Promega, Madison, WI) set to 450 nm with wavelength correction set to 540 nm.

### TUNEL staining for the detection of the end nucleolytic cleavage of chromatin

DNA damage was measured by the TUNEL Apoptosis Detection Kit (Millipore, Temecula, CA) following the manufacturer’s protocol. The lung sections were deparaffinized, rehydrated, and labeled with Avidin-FITC. The slides were sealed with Prolong Gold Antifade Reagent (Invitrogen, containing DAPI) before fluorescence microscopy analysis.

### Co-Immunofluorescence staining of GFP and α-SMA in lung sections of mice administered with PBS or Ad-GFP viruses

5 and 14 days after administration of PBS, bleomycin, and Ad-GFP adenoviruses (Vector Biolabs, Malvern, PA), mice lungs were embedded, sectioned, deparaffinized, rehydrated, and immuno-labeled with the GFP antibody (Living Colors Full-Length GFP Polyclonal Antibody (rabbit), Clontech Laboratories, Inc. Mountain View, CA) and the α-SMA antibody (Sigma (mouse)) and these antibodies were probed with Alexa Fluor-568 and 488 labeled secondary antibodies (Invitrogen), respectively. The nucleolus was counter stained with DAPI (Invitrogen, Grand Island, NY) before immunofluorescence microscopy analysis.

### Statistical analysis

One-way analysis of variance (ANOVA) was used to analyze results. When the results showed a significant difference by the ANOVA, we used the Student’s t-test for further analysis. Statistical significance was set at the 0.05 and 0.01 levels. Results are expressed as mean ± SEM.

## Results

### Intratracheal instillation of Ad viruses causes lung injury and lung inflammation

**Figure 1:**
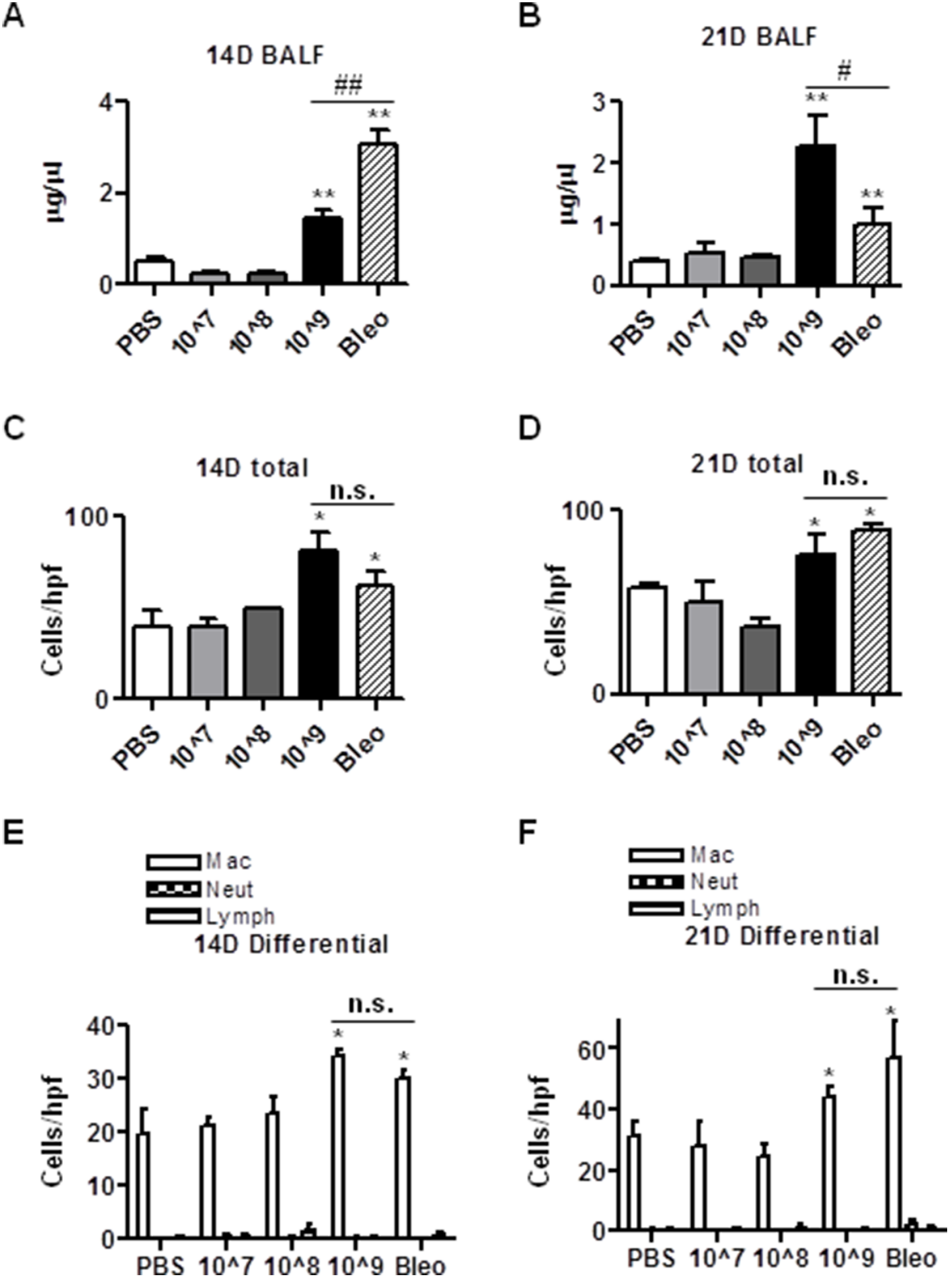
Intratracheal instillation of Ad viral vectors causes lung injury and lung inflammation. C57bl/6 mice were intratracheally instilled with Ad viral vectors at the doses of 1×10^7^/ifu, 1×10^8^ ifu, and 1.625×10^9^ ifu per mouse. PBS and bleomycin were administered as negative and positive controls. 14 and 21 days after administration of PBS, bleomycin and Ad viral vectors, mouse bronchoalveolar lavage fluids (BALF) were collected and used to determine the protein concentration (A and B). Aliquots of 180 µ BALF were applied to cytospin and Wright staining to determine total cell counts and differential cell counts under microscope (200X magnification) (C, D, E, F). The cell counts were expressed as numbers of cells/high power field (hpf). n≥5; * and #, p<0.05; ** and ##, p<0.01; n.s, no significance. * and ** compare the difference between PBS and other groups. #, ##, and n.s. compare the difference between the high dose AD vector group and the bleomycin group.

To investigate whether intratracheal administration of Ad viruses induces lung injury and inflammation, we instilled 50 µl of PBS or PBS containing 10^7^, 10^8^, and 1.625×10^9^ ifu of Ad viruses into the lungs of each mouse. We instilled mice with the same volume of bleomycin (0.045 U/mouse) as the positive control for lung injury and fibrosis. We maintained mice for 14 days and 21 days after the administration of these agents. We then collected the bronchoalveolar lavage fluids (BALF) from the lungs. We measured the protein concentrations in the BALF as an index of lung injury. As shown in Fig. 1A, 14 days after instillation, although lower doses of Ad vectors (10^7^ and 10^8^ ifu/mouse) did not increase protein concentrations in BALF, the high dose (1.625×10^9^ ifu/mouse) increased BALF protein concentration to a significant lesser level than that of bleomycin-treated mouse lung BALF (about half of that of bleomycin-treated mouse lung BALF). However, 21 days after instillation, the mice administered with Ad viruses have sustained significant higher levels of BALF protein concentrations, whereas the protein concentration of BALF in the mice administered bleomycin decreased (Fig. 1B). These results suggest that intratracheal instillation of high dose of Ad vectors induces a modest but sustained lung injury.

Ad viruses are known to activate innate immunity and bleomycin is also known to induce inflammation, concurrently with lung injury. To investigate whether Ad virus administration induces inflammation in lung, we took 180 µl of BLAF from each mouse, spun down the cells to the slides, and stained with Wright stain. We counted the total numbers of cells and the numbers of macrophages (Mac), neutrophils (Neut), and lymphocytes (Lymph) (Fig. 1C–F). We found that instillation of lower doses of Ad viruses did not affect total and differential cell counts in BALF, while instillation of the high dose Ad viruses increased total and differential cell counts to levels comparable to those in mice treated with bleomycin, suggesting that intrachacheal instillation of the high dose of Ad viruses induces lung inflammation.

### Intratracheal instillation of Ad viruses induces lung fibrosis

**Figure 2:**
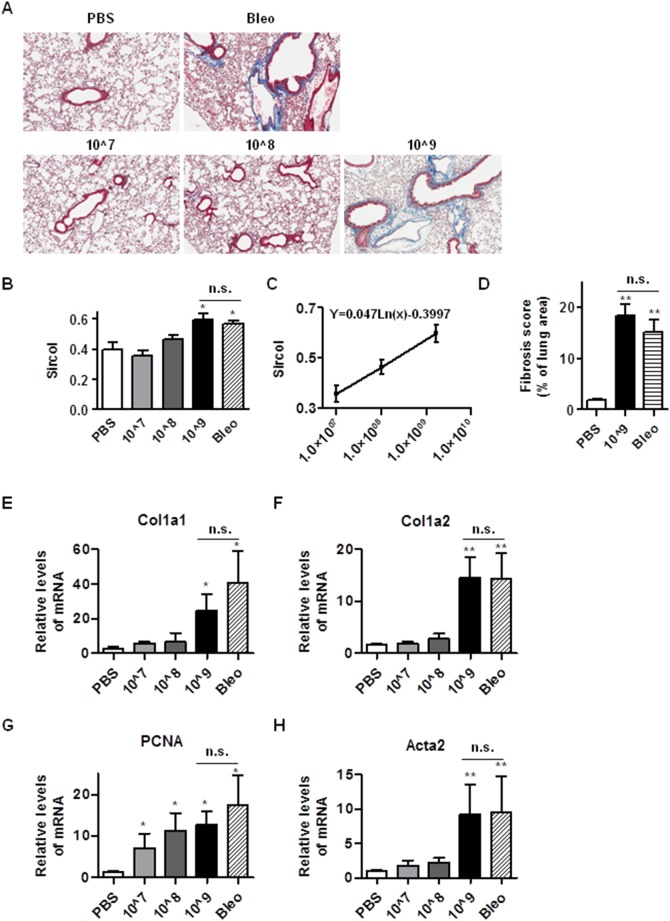
Intratracheal instillation of Ad viral vectors causes pulmonary fibrosis. 21 days after administration of PBS, bleomycin and Ad viral vectors (1×10^7^/ifu, 1×10^8^ ifu, and 1.625×10^9^ ifu per mouse), mouse lungs were harvested, fixed, embedded and sectioned for H&E and Trichrome staining (A). The harvested lungs of these mice were used for collagen analysis by the Sircol assay (B). The content of collagen was normalized to the total amount of protein. We plotted the collagen contents against the natural logarithm of the dose of Ad virus and calculated the equation of the line of the best-fit as indicated in the insert (C). We scan the slides of the Masson’s Trichrome staining on the Aperio ScanScope CS at 20x magnification at standard resolution and measured the percent of tissue area that was classified as fibrosis with Aperio ImageScope v11 software. We presented the score of fibrosis as the percentage of fibrotic tissue area over the total lung tissue area (D). Mouse lungs were homogenized and are used to extract total RNA for the measurement of mRNA levels of mouse Col1a1 (E), Col1a2 (F), PCNA (G), Acta2 (H) by real time qRT-PCR. RPL19 gene was used as internal control for qRT-PCR. n≥5; *, p<0.05; **, p<0.01; n.s, no significance. * and ** compare the difference between PBS and other groups. n.s. compares the difference between the high dose AD vector group and the bleomycin group.

Bleomycin is known to induce lung fibrosis, following the acute lung injury and lung inflammation [10]. Since the instillation of Ad viruses causes comparable lung injury and inflammation to bleomycin (Fig. 1), we set out to study whether instillation of Ad viruses induces pulmonary fibrosis. We instilled three doses of Ad viruses into the lungs of mice intratracheally and maintained these mice up to 21 days. Mice administered with PBS and bleomycin were studied as negative and positive controls. After the experiment, we collected the mouse lungs and stained them with Trichrome for collagen staining. As shown in Fig. 2A, instillation of either bleomycin or the high dose of Ad viruses (1.625×10^9^ ifu/mouse) increased the amount of collagen staining. We then quantified the collagen content in lungs by Sircol assay and found that mice instilled with the high dose of Ad viruses and bleomycin had a similar collagen content, whereas low doses of Ad viruses had comparable collagen contents to lungs of PBS treated mice (Fig. 2B). In order to better understand the kinetics of Ad viruses mediated fibrosis, we plotted the collagen contents against the dose of Ad virus (Fig. 2C). We found that collagen content linearly correlates with the natural logarithm of the dose of Ad vectors and estimated that 1.27×10^8^ ifu/mouse will generate the collagen content equal to the average of that of 1×10^7^ ifu/mouse and 1.625×10^9^ ifu/mouse. To further quantify the fibrosis in these experiments, we scored the fibrosis by quantifying the area of fibrosis over total lung area with Aperio ScanScope CS. As shown in Fig. 2D, our results suggest that instillation of 1.625×10^9^ ifu/mouse and bleomycin induces comparable fibrotic areas in the mouse lungs.

We also determined the mRNA levels of Col1a1 and Col1a2 and found that the transcription of these genes was upregulated in Ad viruses treated mouse lungs (Fig. 2E and 2F). These results suggest that the intrachacheal instillation of high dose of Ad viruses is sufficient to induce lung fibrosis.

During fibrogenesis, there is elevated fibroblast proliferation and differentiation to myofibroblasts. We measured the expression levels of proliferating cell nuclear antigen (PCNA) and α-SMA (Acta2) and found that administration of Ad viruses induced a dose-dependent increase in PCNA, whereas α-SMA mRNA was upregulated significantly only in the mice treated with high dose of Ad viruses and was comparable to that in bleomycin-treated mice (Fig. 2G and 2H). These results suggest that instillation of high dose of Ad viruses induces lung cell proliferation and fibroblast differentiation.

### Intratracheal instillation of Ad viruses increases TGF-β1 production

**Figure 3:**
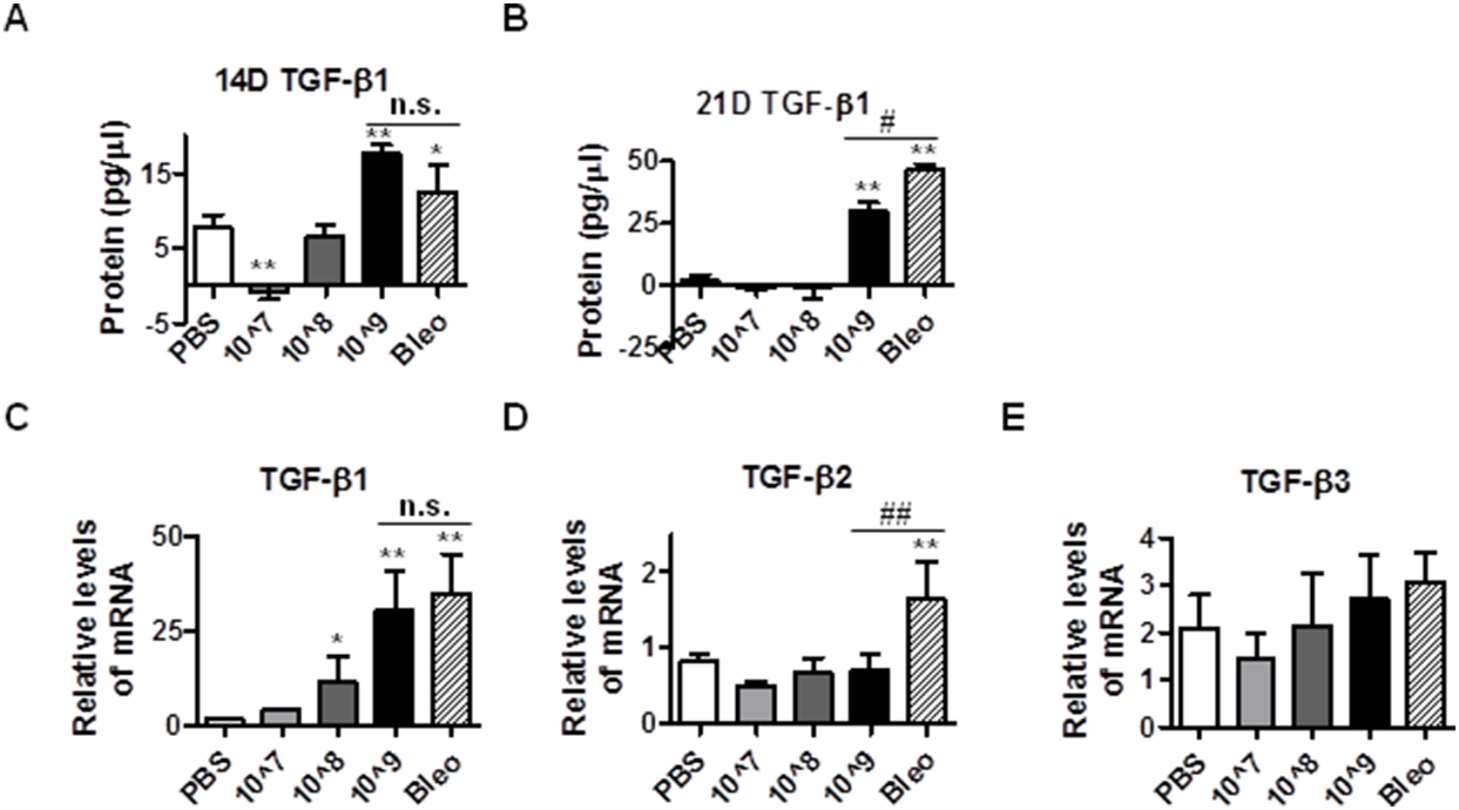
Intratracheal instillation of Ad viral vectors induces TGF-β production in mouse lungs. 14 and 21 days after administration of PBS, bleomycin and Ad viral vectors, mouse BALFs were collected and used to determine the concentration of active TGF-β (A and B). (C–E) 21 days after administration with PBS, bleomycin, and Ad vectors, mice lungs were used to extract total RNA for the measurement of mRNA levels of mouse TGF-β1, TGF-β2, and TGF-β3 by real time qRT-PCR. RPL19 gene was used as internal control for qRT-PCR. n≥5; * and #, p<0.05; ** and ##, p<0.01; n.s, no significance. * and ** compare the difference between PBS and other groups. #, ##, and n.s. compare the difference between the high dose AD vector group and the bleomycin group.

TGF-β1 is a potent fibrogenic cytokine, which plays a critical role in fibrogenesis and is elevated in the bleomycin-induced model of fibrosis. To address whether Ad vectors induce pulmonary fibrosis by induction of TGF-β1, we measured the amount of TGF-β1 in the BALF by ELISA. As shown in Fig. 3A and Fig. 3B, at 14 and 21 days post instillation, treatment with Ad vectors and bleomycin significantly increased the amount of TGF-β1 in BALF. Bleomycin treated mice appeared to have higher levels of TGF-β1 in BALF than mice instilled with Ad vectors 21 days post-instillation. We also measured the mRNA levels of three members of TGF-β family and found that TGF-β1 was upregulated in both bleomycin and Ad vector groups (Fig. 3C), that TGF-β2 is only upregulated by bleomycin (Fig. 3D), and that treatment with neither bleomycin nor Ad vectors affected TGF-β3 mRNA levels (Fig. 3E). These results suggest that instillation of Ad vectors also likely induces fibrosis by increasing TGF-β production.

### Intratracheal instillation of Ad viruses increases expression of profibrotic cytokines

**Figure 4:**
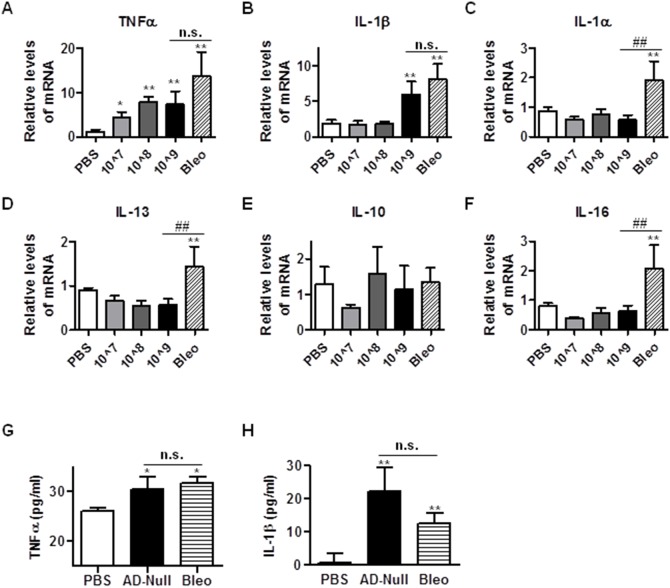
Intratracheal instillation of Ad viruses increases expression levels of profibrotic cytokines. (A–F) 21 days after administration with PBS, bleomycin, and Ad vectors, mice lungs were harvested and were used to extract total RNA for the measurement of mRNA levels of mouse TNFα (A), IL-1β (B), IL-1α (C), IL-13 (D), IL-10 (E), and IL-16 (F) by real time qRT-PCR. RPL19 gene was used as internal control for qRT-PCR. (G–H) 21 days after administration of PBS, bleomycin and high dose Ad viral vectors (1.625 ifu/mous), mouse BALFs were collected and used to determine the concentration of TNFα (G) and IL-1β (H). n≥5; *, p<0.05; ** and ##, p<0.01; n.s, no significance. * and ** compare the difference between PBS and other groups. ## and n.s. compare the difference between the high dose AD vector group and the bleomycin group.

Pulmonary fibrosis is associated with elevated levels of inflammatory cytokines and chemokines, which contribute to disease progression [15]. Numerous reports suggest that in the bleomycin-induced fibrosis model, IL-1α, IL-1β, TNFα are upregulated [15]–[18]. Consistently, our results also suggest that instillation of Ad vectors induces TNFα in a dose dependent manner (Fig. 4A). Instillation of Ad vectors also induced IL-1β (Fig. 4B) but not IL-1α (Fig. 4C), whereas bleomycin treatment induced both IL-1α and Il-1β (Fig. 4B and 4C).

IL-13 is a profibrotic cytokine and can cooperate with TGF-β [15]. In the bleomycin-induced fibrosis model, IL-13 was elevated as expected [19], [20]. However, instillation of Ad vectors did not affect the expression of IL-13 (Fig. 4D).

IL-10 is an anti-inflammatory cytokine that can suppress TGF-β production and procollagen synthesis, thereby inhibiting fibrosis. Previous reports suggest that bleomycin does not alter IL-10 expression [15], [21]. Our study showed that the instillation of neither bleomycin nor Ad vectors altered the expression of IL-10 (Fig. 4E).

IL-16 is a pro-inflammatory cytokine, which serves as a chemoattractant for CD4^+^ T-lymphocytes, monocytes, eosinophils, and dendritic cells [22]. It has been shown that IL-16 expression is elevated in both murine and human fibrosis, therefore, IL-16 is a candidate biomarker for IPF [22]. Our results show that although bleomycin treatment did induce the expression of IL-16, instillation of Ad vectors had little effect on the expression of IL-16 (Fig. 4F).

Since TNFα and IL-1β are two main cytokines induced after Ad vector instillation, we measured the amount of TNFα and IL-1β in the BALF, collected from mice 21 days post instillation, by ELISA. As shown in Fig. 4G and Fig. 4H, treatment with Ad vectors and bleomycin significantly increased the amount of TNFα and IL-1β in BALF. Ad vector treated mice appeared to have higher levels of IL-1β in BALF than mice instilled with bleomycin, whereas TNFα levels are comparable between belomycin and Ad vector group.

### Intratracheal instillation of Ad viruses causes dysregulation of ECM proteins and MMPs

**Figure 5:**
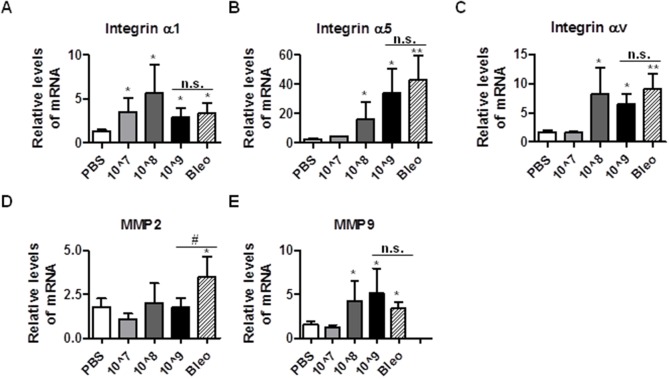
Intratracheal instillation of Ad viruses causes dysregulation of ECM proteins and MMPs. 21 days after administration with PBS, bleomycin, and Ad vectors, mice lungs were harvested and were used to extract total RNA for the measurement of mRNA levels of mouse Integrin α1 (A), Integrin α5 (B), Integrin αv (C), MMP-2 (D), and MMP-9 (E) by real time qRT-PCR. RPL19 gene was used as internal control for qRT-PCR. n≥5; * and #, p<0.05; **, p<0.01; n.s, no significance. * and ** compare the difference between PBS and other groups. # and n.s. compare the difference between the high dose AD vector group and the bleomycin group.

One of the hallmarks of fibrosis is the accelerated deposition of extracellular matrix (ECM), which alters the matrix-integrin receptor signaling to regulate fibroblast behavior [13]. Therefore, we measured the mRNA levels of integrin α1 (collagen receptor), α5 (fibronectin receptor), and αv (activation of TGF-β). We found that administration of Ad vectors, as well as bleomycin, induced expression of integrin α1, α5, and αv (Fig. 5A–C). Although induction of integrin α1 occurred after the administration of the lowest dose of Ad vector, integrin α5 and αv were upregulated only after the administration of higher doses of Ad vectors (10^8^ and 1.625×10^9^ ifu/mouse). These results suggest a selective regulation of ECM receptors by the instillation of Ad vectors. ECM is controlled by the metalloprotease (MMPs), including MMP2 and MMP9, which are also known to contribute to fibrosis [23], [24]. To address whether instillation of Ad vectors causes the deregulation of MMPs, we measured the mRNAs of MMP2 and MMP9 in mouse lungs instilled with PBS, Ad vectors, and bleomycin. We found that although bleomycin induced expression of both MMP2 and MMP9, administration of Ad vectors only induced expression of MMP9 (Fig. 5D and 5E). These results suggest that there may be a unique ECM fingerprint in Ad-induced fibrosis.

### Intratracheal instillation of Ad viruses induces expression of Wnt signaling genes that are associated with pulmonary fibrosis

**Figure 6:**
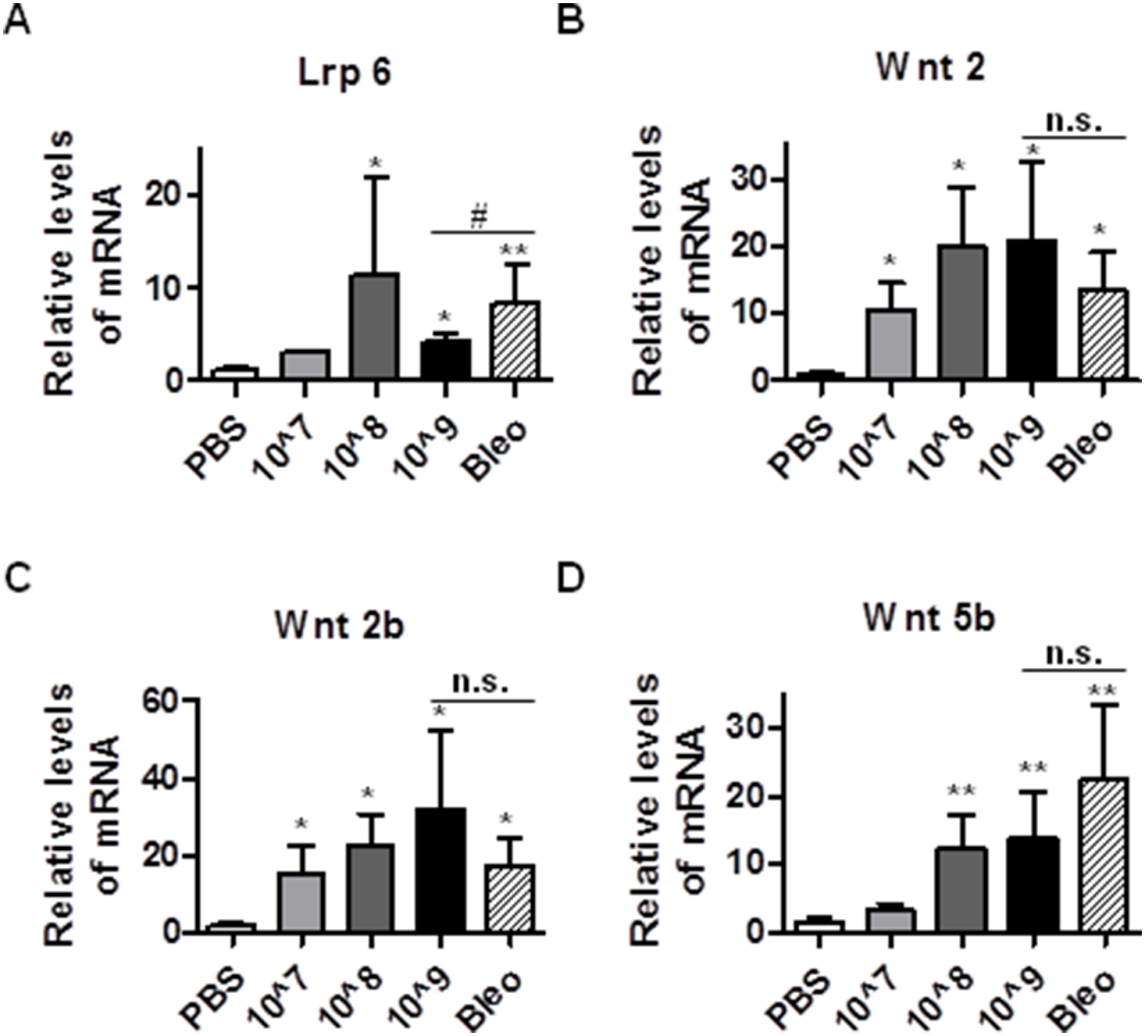
Intratracheal instillation of Ad viruses causes elevated expression of Wnt signaling genes associated with fibrosis. 21 days after administration with PBS, bleomycin, and Ad vectors, mice lungs were harvested and were used to extract total RNA for the measurement of mRNA levels of mouse Lrp6 (A), Wnt2 (B), Wnt2b (C), and Wnt5b (D) by real time qRT-PCR. RPL19 gene was used as internal control for qRT-PCR. n≥5; * and #, p<0.05; **, p<0.01; n.s, no significance. * and ** compare the difference between PBS and other groups. # and n.s. compare the difference between the high dose AD vector group and the bleomycin group.

**Figure 7:**
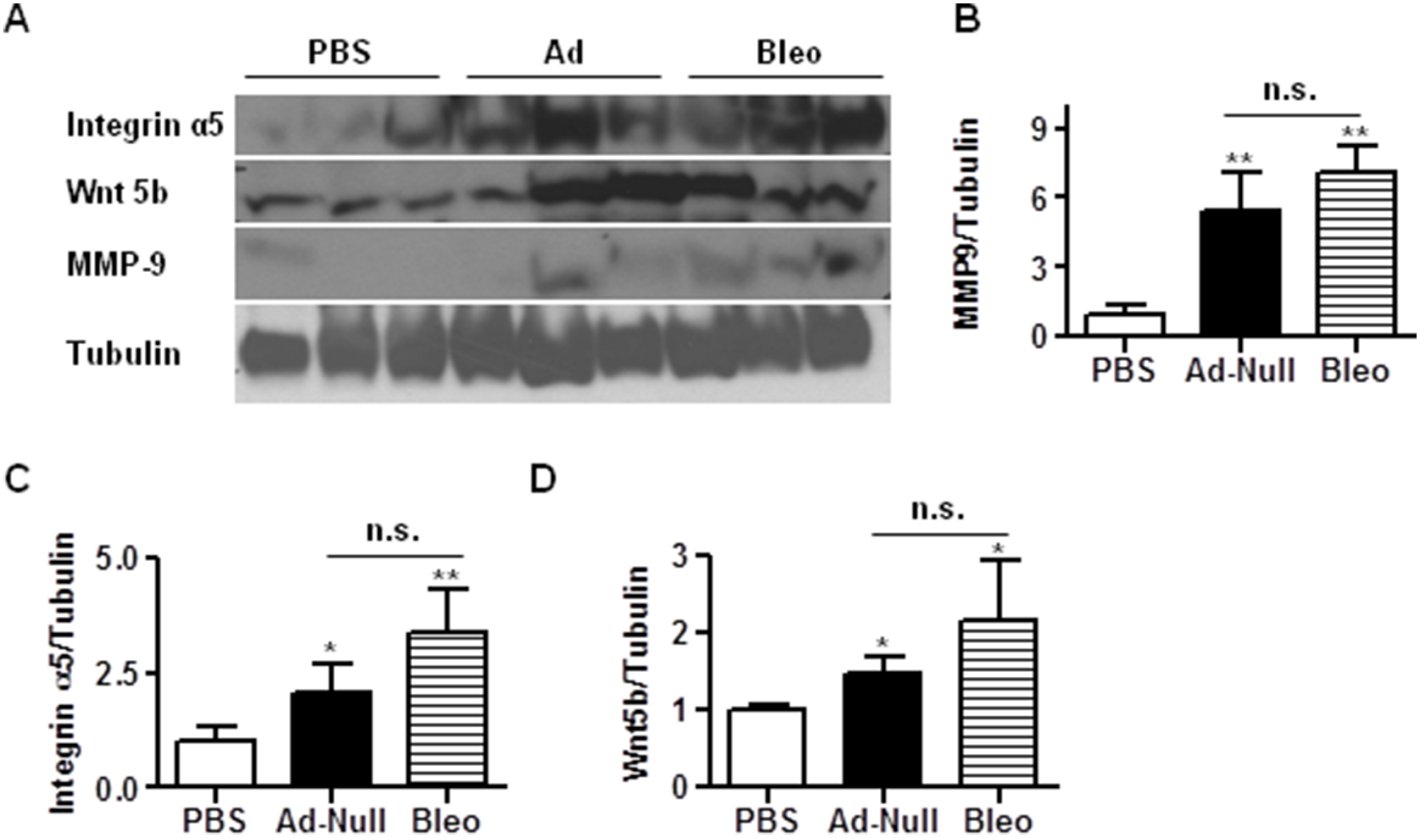
Intratracheal instillation of Ad viruses induces cytokine levels in BALF and protein expression of ECM and Wnt proteins. 21 days after administration of PBS, bleomycin and high dose Ad viral vectors (1.625 ifu/mous), mice lungs were homogenized for the measurement of protein levels of Integrin α5, Wnt5b, and MMP-9 by Western blot analysis. The amount of tubulin was used as control for equal loading. The representative blots are shown in (A) and the quantification of relative expression of MMP-9, Integrin α5 and Wnt5b are shown in B, C, and D, respectively. n≥5; *, p<0.05; **, p<0.01; n.s, no significance. * and ** compare the difference between PBS and other groups. n.s. compares the difference between the high dose AD vector group and the bleomycin group.

The Wnt proteins are part of a family of secreted molecules regulating cell proliferation [25], [26]. In the β-catenin-dependent canonical pathway, Wnt proteins bind to their cell surface receptor Frizzled (Fz) and the LDL-receptor related protein (Lrp) [27], [28]. The Wnt pathway is known to play a critical role in fibrogenesis by inducing expression of their downstream target genes [29]–[33]. IPF patients express elevated levels of Wnt2b, 5b and Fz-related protein as well as β-catenin, proteins known to promote fibrosis or cell proliferation [30]–[32]. Additionally, type II alveolar epithelial cells and myofibroblasts in the distal airway and fibrotic foci in samples from IPF patients displayed nuclear accumulation of β-catenin, which promotes fibroblast proliferation and migration [29], [34]. To investigate whether Wnt signaling is altered in Ad-mediated fibrosis, we measured the expression levels of selected Wnt signaling molecules. Our results showed that administration of Ad vectors induced expression of Lrp6, Wnt2, 2b, and 5b in a dose dependent manner (Fig. 6A–D). Although induction of Lrp6 was less in Ad vectors-induced fibrosis than bleomycin-induced fibrosis (Fig. 6A), induction of Wnt2, 2b, and 5b was comparable between these two fibrosis models (Fig. 6B–D). To further corroborate these results, we measured the protein levels of integrin α5 and MMP9 in lungs of mice treated with PBS, high dose Ad vector (1.625×10^9^ ifu/mouse), and bleomycin. As shown in Fig. 7A–C, bleomycin and instillation of high dose Ad vector induced expression of integrin α5 and MMP9 protein in comparable levels. We also measured the protein levels of Wnt5b in lungs of mice treated with PBS, high dose Ad vector (1.625×10^9^ ifu/mouse), and bleomycin. As shown in Fig. 7A and 7D, bleomycin and instillation of high dose Ad vector induced expression of Wnt5b protein in comparable levels. These results suggest that instillation of Ad vector may also induce Wnt signaling to promote fibrosis.

### Intratracheal instillation of Ad viruses induces DNA damages

**Figure 8:**
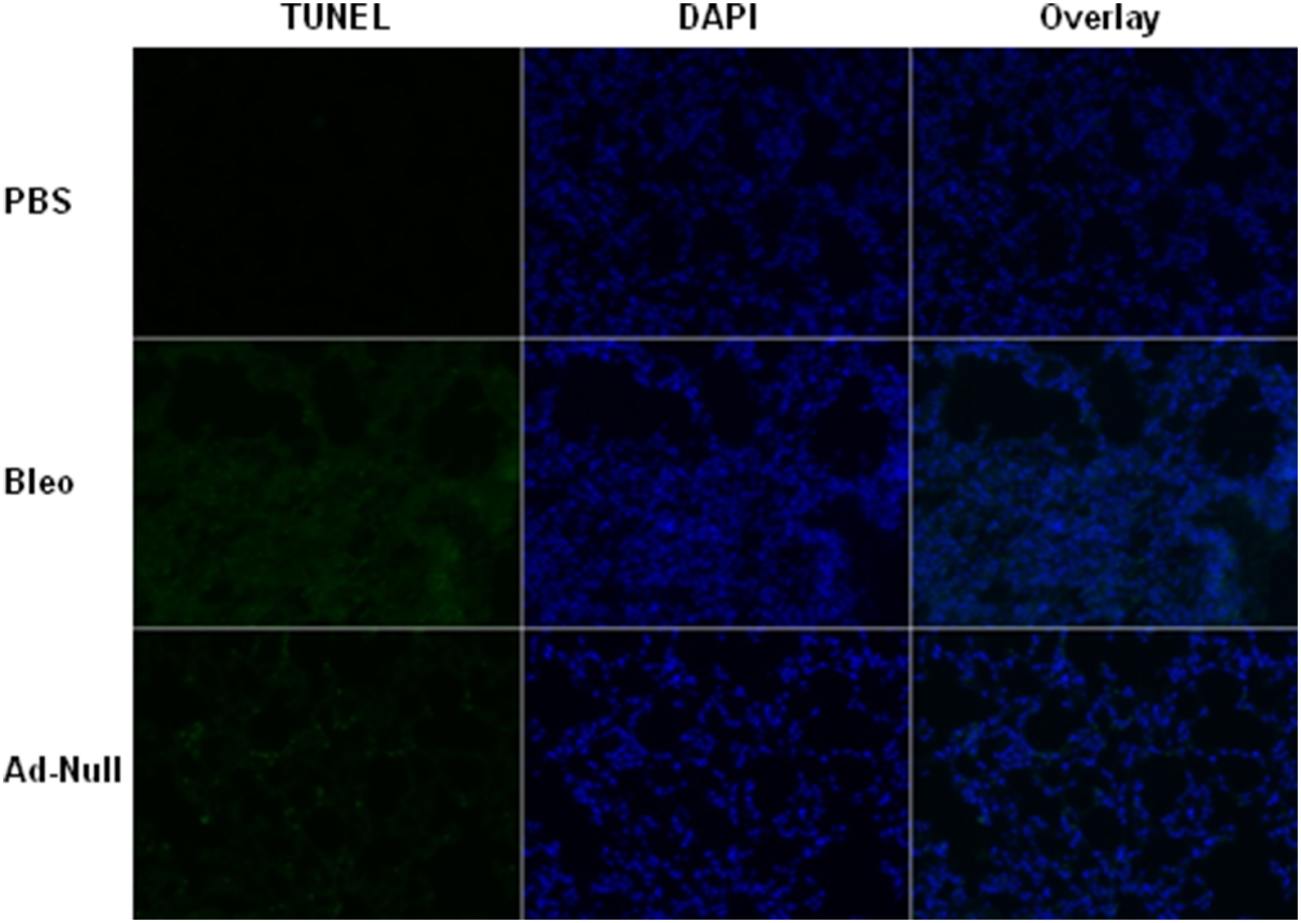
Intratracheal instillation of Ad viruses induces DNA damages. 21 days after administration of PBS, bleomycin and high dose Ad viral vectors (1.625 ifu/mous), mouse lungs were harvested, fixed, embedded and sectioned for TUNEL staining (green color) and DAPI counterstaining (blue color). Fluorescence microscopy images were taken with 10X objective lens.

Bleomycin is known to induce DNA damage and alveolar epithelial injury, which is part of initiating stimuli for pulmonary fibrosis. To address whether administration of high dose Ad vectors induces DNA damage, we measured the DNA damage by TUNEL staining. As shown in Fig. 8, mouse lungs administered with PBS exhibited minimal TUNEL signal, lungs administered with either bleomycin or Ad vectors showed strong TUNEL staining, suggesting that administration of Ad vectors also causes DNA damage.

### Ad viral infection-mediated fibrosis is not limited to the infection sites

**Figure 9:**
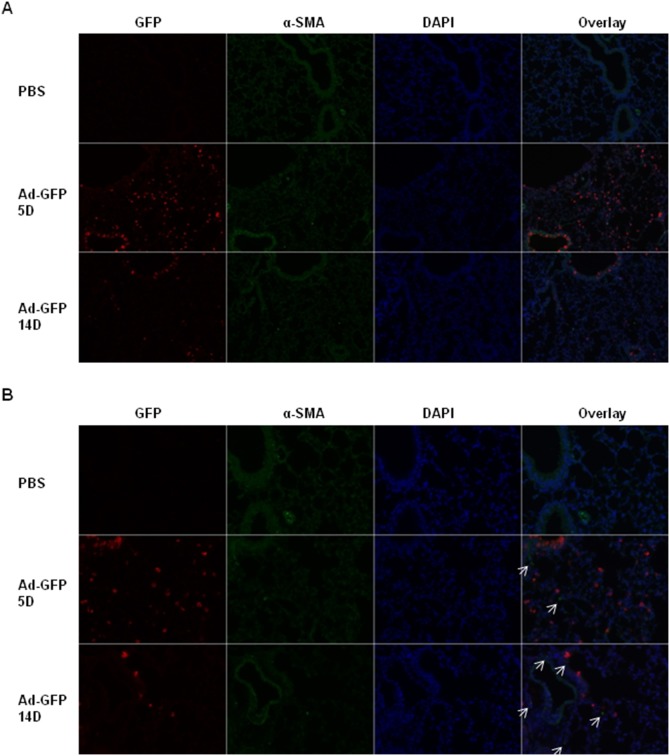
Ad viral infection-mediated fibrosis is not limited to the infection sites. C57bl/6 mice were intratracheally instilled with PBS and Ad-GFP viral vectors at the doses of 1.625×10^9^ ifu per mouse. 5 (5D) and 14 (14D) days after administration of PBS and Ad-GFP viral vectors, mouse lungs were harvested, fixed, embedded and sectioned for co-immunofluorescence staining for GFP (red color) and α-SMA (green color) and DAPI (blue) counterstaining. Fluorescence microscopy images were taken with 10X objective lens (A) and 20X objective lens (B). White arrows indicate the position of α-SMA-positive cells.

To investigate whether Ad vector infection sites co-exist with the fibrotic sites, we administered mice with Ad-GFP vectors and co-stained GFP with α-SMA (for myofibroblasts). Since a previous report has shown that the Ad-vector mediated gene expression is transient and disappears after 7–14 days [35], we first determined the timing of GFP expression in mouse lungs after Ad-GFP viral vectors administration. We found that intratracheal delivery of Ad-GFP generated robust GFP expression in airway, alveoli, but not in blood vessels 5 days after administration (Fig. 9A). However, 14 days after administration, the number of GFP positive cells was significantly less (Fig. 9A). Although the maximal fibrosis occurs in 21 days postinfection, there is significant lung inflammation and collagen induction 14 days postinfection (Figs. 1–3). Thus we chose to test whether the infection sites co-exist with the fibrotic sites up to 14 days postinfection. We found that GFP-positive and α-SMA-positive cells did not co-localize (Fig. 9B). However, 5 days after administration, the GFP-positive cells were at the proximity of α-SMA-positive cells (Fig. 9B, middle panel). 21 days after administration, many GFP-positive cells were distant from α-SMA-positive cells and the number of from α-SMA-positive cells was significantly increased (Fig. 9B, lower panel). These results suggest that the fibrosis is not limited to the infection sites.

## Discussion

Although it is known that Ad vectors activate innate and adaptive immunity and inflammation contributing to the pathogenesis of fibrosis, it is unclear whether intratracheal administration of Ad vectors is sufficient to induce lung injury and lung fibrosis. Our studies suggest that intrachacheal administration of a high dose of Ad vectors induces lung inflammation, lung injury, and pulmonary fibrosis. Thus, our finding cautions researchers on the interpretation of their data when they use adenovirus.

Our studies suggest that administration of Ad vectors induces lung injury, inflammation, and fibrosis in a dose dependent manner (Figs. 1–3). Instillation of lower doses of Ad vectors (10^7^ ifu/mouse and 10^8^ ifu/mouse) elicits minimal lung injury, inflammation, and fibrosis, whereas instillation of the high dose is sufficient to induce similar effects, as in bleomycin-induced fibrosis in mice (Figs. 1–3). This dose dependent response is consistent with previous reports that Ad vectors induce a sustained and dose dependent inflammation [1], [36]. In gene therapy, Ad vectors associated inflammation has been reported to limit expression levels and duration of expression of the target gene, which can be overcome by increasing dose of Ad vector [4], [36]. However, our study suggests that there is a limit to how high we can raise the dose of Ad vectors because administration of the high dose Ad vectors induces significant inflammation, injury and fibrosis.

Ad vectors have been used to overexpress TGF-β1 to induce fibrosis [3], [12]. In these studies the Ad vectors were used in relatively lower doses and TGF-β1 expression was transient and sustained for only 3–5 days, whereas the degree of fibrosis was maximal 28 days after administration. In contrast, bleomycin induces optimal fibrosis in 21 days. Thus, it is possible that Ad vector and TGF-β1 expression may synergistically enhance and prolong the inflammation signal to cause fibrosis. Accordingly, our results show that instillation of Ad vectors induces TGF-β1 production and mRNA expression of TGF-β1 but not TGF-β2 or TGF-β3 (Fig. 3). More importantly, adenoviral E1A DNA is detected in IPF patients and Ad viruses are known to exacerbate COPD patients [5], [37]. Therefore, sustained inflammation after instillation of Ad vectors may potentiate pulmonary fibrosis.

Many pro-fibrotic cytokines such as TNFα and IL-1β are upregulated in fibrotic lungs [38], [39]. We found that both Ad vectors and bleomycin induced expression of TNFα and IL-1β (Fig. 4A, 4B, 4G, and 4H). IL-1α, which is cell surface-bound and rarely secreted, is known to regulate IL-6 and is key to bleomycin-induced fibrosis [40], [41]. We found that IL-1α is induced in bleomycin but not Ad vector mediated fibrosis (Fig. 4C). Thus, IL-1α/IL-6 axis may be functioning in bleomycin but not Ad vector mediated fibrosis. However, it is worth to note that Ad vector-mediated IL-1α production is beneficial for vaccine development or cancer gene therapy [42], [43].

Cell membrane integrin receptors are known to regulate fibrosis. Integrin αv is key to the activation of TGF-β1 [44] and integrin α5 and α1 are elevated in fibrotic tissues [13]. As expected, we found that administration of Ad vectors induces expression levels of integrin α5, α1, and αv (Fig. 5A–C), consistent with the elevation in TGF-β1 production (Fig. 3). Integrin αv also regulates the entry of Ad virus, thus our results suggest that integrin αv and Ad virus may form a feed forward loop to amplify the viral response. MMPs regulate ECM synthesis and degradation. MMP2 and MMP9 are the subgroup of MMPs that have been most extensively studied in interstitial lung diseases and their genes and proteins are upregulated in IPF [45]. We show that MMP9 is upregulated in both bleomycin and Ad vector-mediated fibrosis, whereas MMP2 is only upregulated in the bleomycin model (Figs. 5D–E, 7A–C).

Emerging data suggest that Wnt signaling may play a role in IPF [46], [47]. IPF patients have elevated levels of Wnt2, Wnt2b, Wnt5b and Fz-related protein known to promote fibrosis or proliferation [30]–[32]. Consistently, we found that administration of high dose of Ad vectors induced expression levels of Lrp6, Wnt 2, Wnt2a, and Wnt5b (Figs. 6 and 7).

One feature of fibrosis is epithelial injury and bleomycin has been shown to cause DNA damage and alveolar epithelial cell death. Interestingly, we show that Ad vectors also cause DNA damage in the mouse lungs (Fig. 8), suggesting a similar epithelial damage in Ad vector- mediated fibrosis. In Ad-GFP viruses infected mouse lungs, GFP-positive cells do not co-localize with α-SMA-positive cells, suggesting that Ad vector infected epithelial cells are unlikely undergoing epithelial-mesenchymal transition (EMT) (Fig. 9A). Ad vectors induces the number of α-SMA-positive cells, and whether these cells are myofibroblasts from interstitial fibroblast activation, EMT or recruitment of circulation progenitors cells warrants further investigation. Although in the injury/inflammation stage (5 days post infection), α-SMA-positive cells are typically in the close proximity of infection sites, in the fibrosis stage (14 days post infection), infection site and fibrotic site can be distant (Fig. 9B). This spaciotemporal pattern suggests that immediately after infection, there is a local response of activation of α-SMA positive cells, whereas later the sustained inflammation amplifies this response to distal areas. This observation is consistent with the sustained inflammation in this model (Figs. 1, 3–4).

Several mouse models of pulmonary fibrosis, including bleomycin, silica, FITC, and irradiation, have been commonly used to investigate the pathophysiology of pulmonary fibrosis [8], [48]. However, none of these recapitulates the pathology of IPF. Similarly, Ad vectors-mediated fibrosis also has its limitations, with strong inflammation and lung injury and the fibrosis in peribronchial region. Furthermore, Ad vectors generate less TGF-β and other profibrotic cytokines than bleomycin (Figs. 3–4), suggesting that it is a modest fibrotic inducer. It is notable that Lagares and colleagues used Ad vectors-mediated gene transfer to investigate the role of ET-1 in bleomycin-induced fibrosis and they injected a dose of 2×10^∧^9 PFU/mouse [35]. Although in their study they used Ad null vectors as control for ET-1 vector and did not compare the effects of Ad null vectors to that of PBS, it does raise an important aspect on the handling of Ad vectors. Ad vector virulence may easily decrease by frequent freeze-thaw thus it is critical to aliquot them and measure virulence before experiments. The method used to measure Ad vector virulence varies and thus the infection efficiency may differ widely between viral particle, pfu, and ifu. Another important question is to address whether Ad vectors-mediated fibrosis is sustainable. Degryse and colleagues reported that repetitive instillation of bleomycin induces a more severe and sustained fibrosis [49], thus we cannot rule out that repetitive administration of Ad vectors may also give rise to better fibrotic features. An equally interesting study will be to investigate whether Ad antibody can prevent or even reverse high titer Ad induced lung injury/inflammation/fibrosis. Furthermore, female rats showed higher mortality rates and more severe fibrosis than did male rats in bleomycine model [50]. It will be valuable to investigate the gender effects in this model. Nonetheless, given the different inflammatory response and distinct expression pattern of ECM and MMP genes between treatments of Ad vectors and bleomycin, Ad vector-mediated fibrosis model may provide an alternative strategy to dissect the distinct role of cytokines in fibrogenesis and further studies are warranted to investigate whether repetitive instillation of Ad vectors induce more severe and sustained fibrosis, whether instillation of Ad vectors induces more severe fibrosis in female mice.
